# Chlor-Acetic Acid in Cancer

**Published:** 1879-05

**Authors:** S. J. Bumstead

**Affiliations:** Decatur, Ill.


### Chlor-Acetic Acid in Cancer.

EDITOR MEDICAL BRIEF:—Although I had frequently read accounts of the value of acetic acid in cancer, it was not until the spring of 1873, that I was personally impressed with its value. Being in Paris, I visited the eye clinic of DeWecker, the celebrated oculist of that city. Among many objects of interest he called our attention to a case of epithelioma of the forehead, which was rapidly disappearing under the use of acetic acid, pricked into the nodules by means of glass rods with fine points. He spoke in a very impressive manner as to the value of this treatment, and of course I did not forget it. I have used it in the same way, and successfully in the epitheliomata, and it may have a more curative effect in other varieties than is now thought to be the case. But good as the above method is I have ascertained by testing, that chlor-acetic acid is much better, more powerful in its action, and yet is easily controlled. The acid is probably better known to aurists, it being used to apply to aural polypi, being very effective, and its action being at once arrested by syringing water into the meatus. Its action here and my knowledge of the value of acetic acid in cancer suggested the use of the other and stronger agent in cancer, and from repeated trials I can conscientiously recommend it to the profession in all cases where a destruction of such is desirable by other means than the knife. It can be applied in the crystal form, when the growth is an external one, and just enough water may be added to render it soluble, and then injected into the tissues. I usually apply the chlor-acetic once every three or four days while destroying the growth, and the strongest acetic acid about twice a day. I am also of the opinion that some incurable cases may be mitigated, and the lethal termination deferred by its use.- -*Medical Brief.* S. J. BUMSTEAD, M. D.

Decatur, Ill., April 3d, 1879.

